# A Multicentric Study to Validate the Patient-Reported Experience Measures (PREM) Tool for Wound Management and Safety in India

**DOI:** 10.7759/cureus.88176

**Published:** 2025-07-17

**Authors:** Jothi C Michael, Malathi Murugesan, V B Narayana Murthy, Selva Seetharaman, Hepsibah Sharmil, Hannah J Rosy, Phalakshi Manjrekar, Shankar Shanmugam Rajendran, Thankam Gomez

**Affiliations:** 1 Health Policy, Healthbridge, Chennai, IND; 2 Microbiology, Meenakshi Mission Hospital and Research Centre, Madurai, IND; 3 Plastic and Reconstructive Surgery, Kauvery Hospital, Chennai, IND; 4 Plastic Surgery, Dr. Selva Plastic Surgery Clinic, Chennai, IND; 5 Institute of Plastic, Aesthetic and Reconstructive Surgery, Gleneagles Global Hospital, Chennai, IND; 6 Department of Neonatology, College of Nursing, Chettinad Hospital and Research Institute, Kelambakkam, Chennai, IND; 7 Pediatrics, Gleneagles Healthcare, Chennai, IND; 8 Cardiac/Thoracic/Vascular Surgery, Hinduja Group Hospitals, Mumbai, Mumbai, IND; 9 Oncology, College of Nursing, Madras Medical College, Chennai, IND; 10 Nursing Administration, Cygnia Healthcare Academy, Delhi, IND

**Keywords:** infection control, patient education, patient reported experience measure, wound dressing safety, wound management

## Abstract

Background: Effective wound management is a vital aspect of healthcare that significantly influences patient morbidity and mortality. While traditional wound care quality assessments focus on clinical outcomes such as healing rates, infection control, and recurrence, patient experience during wound therapy remains an important yet underexplored dimension of care quality.

Objective: This multi-centric prospective study aimed to develop and validate a Patient Reported Experience Measures (PREM) tool specifically designed to capture patient perspectives on safety, communication, infection prevention, and overall care during wound dressing procedures within the Indian healthcare setting.

Methods: Seventeen hospitals across diverse geographic regions and varying sizes in India participated. The PREM tool was developed through a systematic process involving iterative surveys, focus group discussions with healthcare professionals, and pilot testing. The initial tool included 19 items covering communication, infection control, patient education, privacy, and patient identification. After pilot testing with 170 patients, the instrument was refined and administered to 904 patients. Psychometric validation was performed using confirmatory factor analysis (CFA).

Results: The PREM tool demonstrated excellent reliability with a Kuder-Richardson Formula 20 (KR-20) internal consistency coefficient of 0.748. Majority of patients reported positive experiences: 91.3% confirmed name and ID verification prior to dressing, 95.7% received procedure explanations, 100% observed infection control practices, and 97.6% reported maintained privacy. However, only 56.7% received adequate training for home wound care, and 59.1% could demonstrate dressing techniques to healthcare providers, highlighting a need for improved patient education. Enhanced patient adherence to infection control measures reflected increased awareness following the COVID-19 pandemic.

Conclusion: The study underscores the importance of incorporating patient feedback into wound care quality evaluation, complementing traditional clinical outcomes with experiential factors that influence patient satisfaction and adherence. The validated PREM tool offers a structured framework for healthcare providers to assess and enhance the wound care experience, promoting patient-centered care. Future research should focus on cross-cultural validation, demographic diversification, and integration of PREM data with clinical parameters to improve wound care quality comprehensively.

## Introduction

Wounds form an important aspect in healthcare delivery. Treating them effectively leads to decreased morbidity and sometimes mortality, resulting in better outcomes [1]. Patients, their relatives, and healthcare professionals are all directly or indirectly involved throughout the treatment process, each potentially having different experiences and perspectives on the care provided [2]. The final outcome of treatment may vary, but the patient's experience during the treatment process remains a crucial component of overall care, patient experience during treatment is an important component in the treatment. Patient experience involves aspects of the procedure or the process of wound care delivery itself, in addition to the actual wound care provided by the healthcare professional [3]. Traditional parameters of quality in wound management are based on objective healing outcomes indices like healed wounds, recurrence rates, and ulcer-free days, morbidity data like infection and sepsis [4]. Although many of these outcome variables can be readily obtained from routinely collected administrative data, their use as quality metrics is complicated by risk adjustment challenges. Therefore, an indirect parameter based on direct patient experience of the wound care delivery itself, with the whole process of wound dressings, is considered here to monitor treatment [5]. This is evidenced by Kline et al. [6] that patient-reported experience measures (PREMs) assess the quality of care from the patient's perspective, enhancing patient-centered care and facilitating patient engagement. Additional evidence from multifactorial studies on wound management highlights high satisfaction with interprofessional care services and practical cooperation among professionals. Consequently, it has been concluded that incorporating PREM in clinical practice is essential for integrating patient perspectives [6].

PREM employs a tool developed by healthcare experts in wound management. The validity of this tool is essential for the study, as it measures patient responses to wound care provided. Previous research highlights factors such as dressing choice, healthcare provider skill, and dressing change frequency, which significantly impact patient perceptions and healing outcomes [7]. By examining patient feedback, we can identify strengths and areas needing improvement in wound care practices, ensuring both clinical efficacy and patient comfort. This study aims to gather patient-guided responses, providing valuable insights for healthcare providers and informing future guidelines to enhance patient-centered care in wound management [8]. The primary objective of this study is to develop and validate a structured tool for healthcare personnel to evaluate PREM in wound management and to assess the tool's effectiveness in measuring patient experience and safety in wound care.

## Materials and methods

This multiphase prospective questionnaire development and validation study was conducted from September 2023 to April 2024. The study protocol was approved by the Ethics Committee of Gleneagles Health City, Chennai (Reference number: BMHR/2024/0072), which was chosen as the main ethics committee by the participating centers. The ethical committee approval process involved representation from all 19 participating hospitals. This multi-centric study was conceptualized, coordinated, and executed under the guidance and leadership of the Consortium of Accredited Healthcare Organizations (CAHO), leveraging its national network of accredited hospitals committed to quality and patient safety. Participating institutions were selected based on CAHO’s member network, and all data collection, training, and monitoring processes were standardized through CAHO’s central research team.

Objectives

The aim of this study is to develop and validate a PREM tool specifically designed to assess patient perspectives on wound care procedures within the Indian healthcare context. The objective is to evaluate key aspects of the wound care process, including safety, communication, infection prevention, patient education, and privacy, from the patient's point of view. The study also seeks to determine the tool's effectiveness in measuring the quality and safety of care during dressing procedures and to identify areas for improvement in patient education, particularly in home wound care practices. By developing this tool, the study aims to contribute to enhancing patient-centered care and providing healthcare providers with a structured method for incorporating patient feedback into wound management practices. Additionally, the research seeks to ensure the validity and reliability of the PREM tool through psychometric validation, which will ultimately help improve overall care quality and patient outcomes in wound management.

Study population

The study was conducted in 17 hospitals across India. These hospitals were chosen by purposive sampling, with a mix of small (< 100 beds) medium (100-300 beds) and large hospitals (> 300 beds) across North, South, East, West and Central India (North- 03 South- 05, East- 05, West- 04 and Central- 02).

Inclusion Criteria

Adult patients with wound dressing (any region of the body), conscious and stable, able to give feedback, able to understand, read, and write English, willing to participate in the study. Dressing could be done at the Outpatient Department (OPD), clinics or the Inpatient Department (IPD). Dressing could be done by a doctor, nurse, or qualified wound care technician.

Exclusion criteria included paediatric patients (children less than 18 years), any other procedure associated with dressing, adult patients who come second time for dressing/repeated dressing and pregnant women.

Study procedure

Phase 1: PREMs Tool Design

A team of subject experts, which included plastic surgeons, surgeons, nurses, quality professionals, and healthcare administrators, was chosen from a mix of small and large private, government, and teaching hospitals to ensure representation from across the country. This team participated in two focus group discussions via videoconferencing (VC), to finalize the standard wound dressing procedure related to safety in patients undergoing the procedure. The focus group discussion through VC was moderated by a senior nurse administrator and a plastic surgeon. The moderator prepared a set of points and procedures that were non-directive but controlled the discussion within the frame of the topic and domains. The content was analysed by playing the recorded video and discussion points were gathered. This process identified 19 items that were related to the study domains. Utilizing this, initially a 19-item self-administered questionnaire with instructions in English was built on the wound dressing procedure, to be answered by the patients or patient attenders, based on their care experience. The questionnaire covered the domains of wound dressing safety, patient identification, patient communication, infection prevention, financial awareness, clinical care, and patient privacy. The order of the questions and the response options were also finalized.

An online survey with 30 subject experts and seven patients, from a mix of small and large private, government and teaching hospitals, commented on the understandability and relevance of each item in the questionnaire with a ‘yes’ or ‘no’ response, along with open-ended comments and suggestions to improve the tool. The use of medical jargon was identified, and the domain or category of the question was verified. Face and content validation to assess relevance and understandability of the items was done. This was followed by another focus group discussion involving the core team, which led to item refinements and additions based on the expert opinion. This iteration of the questionnaire had 19 items (Appendix).

Phase 2: Pilot Study

A pilot study was done across 17 hospitals with a sample size of 170. This was the upper limit for sample size based on general recommendations. The questionnaire did not need any modification based on the evaluation of the pilot study results using exploratory factor analysis.

Phase 3: Field Study

The minimum sample size for the field study to assess reliability and validity of the questionnaire, based on the item per participant ratio of 1:10 principle, was 170 [2]. But for a better validity, we proposed that each participating hospital be asked to recruit a minimum of 50 subjects to complete the questionnaire. The questionnaire was completed by 904 participants across the 17 hospital, after informed consent. The psychometric validation testing was done using confirmatory factor analysis (CFA). 

Statistical analyses

Face validity was assessed using expert opinion as described in the methodology. Content validity index (CVI) was calculated using three indices, namely, item level (I-CVI), scale-level (S-CVI), and content validity ratio (CVR) for each item’s relevance.

The I-CVI is the proportion of experts stating the relevance. The S-CVI was calculated using two methods [4], namely S-CVI/UA, the proportion of the items stated as relevant by all the experts (universal agreement (UA) by experts) divided by the total number of items and S-CVI/Ave, the sum the I-CVI scores of all items divided by the total number of items (average of the I-CVI scores for all items across all experts).

The CVR was calculated by dividing the subtraction product of the number of experts indicating an item as relevant and half the number of experts, divided by half the number of experts (CVR=(ne-N/2)/(N/2)) [5,6].

The assessment of the interclass correlation in the pilot study was done using Bartlett's test of sphericity and the sample adequacy was assessed using the Kaiser-Meyer-Olkin measure. After the assumptions related to intercorrelation were satisfied, exploratory factor analysis (EFA) was done using principal component factor analysis (PCA) to extract the factors. Factors with eigenvalue >1.0 were taken as factor models [7,8].

CFA was then conducted to test the results obtained from the EFA by determining the goodness‑of‑fit using the Chi‑square statistic. The following fit indices were utilized for the evaluation and comparison of descriptive goodness‑of‑fit: comparative fit indices (CFIs); root mean square error of approximation (RMSEA); and standardized root mean square residual (SRMR). The acceptable cut‑off for fit indices was indicated by CFI ≥0.90, SRMR ≤0.10, and RMSEA ≤0.08 [9].

## Results

Phase 1

Regarding the person who filled out the questionnaire, the majority were patients themselves (130, 51.2%), followed by bystanders (47, 18.5%) and family members (32, 12.6%). Information on this variable was missing for 45 participants (17.7%) (Table 1).

**Table 1:** Demographic characteristics of the sample (n=254) IMS: Institute of Medical Sciences, KIMS: Krishna Institute of Medical Sciences

Variables	n (%)
Age (years); Mean (SD)	48.84 (17.93)
Sex	
Male	157 (61.8)
Female	97 (38.2)
Hospital Name	
Aureus Hospital	14 (5.5)
Bharati Vidyapeeth Medical College & Hospital	15 (5.9)
Bombay Hospital	15 (5.9)
Chettinad Hospital & Research Institute	15 (5.9)
Fortis Hospital, Kolkata	15 (5.9)
Gleneagles Health City	15 (5.9)
Hannah Joseph Hospital	15 (5.9)
IMS & SUM Hospital	15 (5.9)
Institute of Neurosciences	15 (5.9)
Kauvery Hospital	15 (5.9)
KIMS Kingsway Hospital, Nagpur	15 (5.9)
Metas Adventist Hospital	15 (5.9)
MGM Muthoot Medical Centre	15 (5.9)
My hospital	15 (5.9)
Peerless Hospital	15 (5.9)
Rukmani Birla Hospital	15 (5.9)
Sri Sai Hospital	15 (5.9)
Filled by? (Patient/Bystander/Family)	
B	47 (18.5)
F	32 (12.6)
P	130 (51.2)
Missing	45 (17.7)

A significant majority (91.3%) reported that the healthcare team verified their name and hospital registration number prior to starting the dressing and 95.7% affirmed that the procedure was explained beforehand. Nearly all participants (99.2%) were informed about potential discomfort or pain during the dressing and 97.6% stated that adequate privacy was ensured. Concerning the removal of personal jewellery, 83.9% responded affirmatively, while 14.6% marked it as not applicable. Regarding hand hygiene, 98.8% confirmed that handwashing or use of sanitizer was performed. All participants (100%) acknowledged that the team took proper infection control precautions. Additionally, 90.9% reported that the doctor examined the wound and explained the management plan. However, opinions were nearly divided on whether dressing materials were placed on the patient's bed, with 51.6% saying yes and 48.4% no. Almost all participants (99.6%) confirmed that waste materials were properly discarded and 97.6% felt they were able to clarify doubts with the healthcare team. A total of 98% were advised not to touch their dressing or wound. Among those advised to change dressings at home, 56.7% were educated on how to do so, and 59.1% reported demonstrating the technique to a healthcare provider. Furthermore, 94.9% were instructed to watch for wetness or soakage in the dressing, and 93.3% were advised to monitor for fever as a possible sign of infection. Most participants (97.2%) felt their concerns were adequately addressed and 94.5% were informed about when to seek urgent care. Lastly, 93.3% of respondents knew whom to contact for urgent wound-related issues (Table 2). 

**Table 2:** Summary statistics of study questions

Q.No	Question	Yes	No	NA
1	Did the team verify your name and hospital registration number before starting the wound dressing?	232	19	3
2	Did the doctor/nurse explain the dressing procedure before starting?	243	11	3
3	Were you explained that you will face discomfort/ pain during the change of dressing?	252	2	0
4	Did the team ensure that you had enough privacy before starting the dressing?	248	6	0
5	Did the doctor/nurse/wound care technician remove jewelry (watch/ rings/ bangles/ threads) before dressing your wound?	213	4	37
6	Did the doctor/ nurse wash their hands/ use hand sanitizer before dressing your wound?	251	0	3
7	Did the team take enough precautions to prevent infection, such as wearing gloves, use sterile instruments etc.	254	0	0
8	Did the doctor examine the wound and explain to you the plan of management?	231	23	0
9	Was the dressing material used for dressing kept on your bed?	131	123	0
10	Were the materials used discarded in the waste bins immediately after the dressing?	253	1	0
11	Were you able to clarify/ ask questions to the doctor/ nurse related to wound or its care?	248	6	0
12	Were you advised that, you must not touch your dressing/ wound?	249	5	0
13	If you were advised to change dressing at home, were you/ your family educated on how to change the dressing?	144	37	73
14	If you have been educated, did you or a family member demonstrate how to do the dressing to the doctor/nurse/wound care technician?	150	37	67
15	Were you advised that any wetness/ soakage in the wound is not a good sign and you must watch for any wetness/ soakage in the dressing?	241	13	0
16	Were you advised to watch for fever, as that may be due to infection in your wound?	237	17	0
17	Have all your doubts about wound care been cleared by the team members?	247	6	1
18	Have you been advised when to seek urgent care?	240	13	1
19	Do you know whom to contact to take urgent care in case of need?	237	17	0

Kuder-Richardson coefficient of reliability (KR-20) for a particular set of data is shown in Table 3. It shows that the total number of questions included in the analysis is 19. The internal consistency, measured using the KR-20, was found to be 0.7480. This value falls within the "acceptable" range of reliability, as indicated by the interpretation provided. This suggests that the set of questions exhibits a moderate level of internal consistency and is deemed suitable for analysis. 

**Table 3:** Kuder-Richarson coefficient of reliability (KR-20)

Total Number of Questions	Internal consistency	Interpretation
19	0.7480	Acceptable

## Discussion

This study aimed to create and validate a specialized tool, the PREM, for evaluating wound management within the Indian healthcare system. The PREM tool provides crucial insights into enhancing wound care by incorporating patient feedback on safety and experiences during wound dressing procedures. It proved highly reliable and valid in capturing the essential aspects of wound management, including safety, communication, infection control, and patient education. The study included 904 participants from 17 hospitals, predominantly male (59%), with an average age of 50.8 years. This age demographic reflects the higher prevalence of chronic wounds in older adults [9]. It was observed that PREM research is largely concentrated in the USA, the Netherlands, and England, but has not spread across developing and underdeveloped nations. This also explains the predominance of the English language in PREM scales. The availability and usability of PREM instruments in diseases/conditions and specific populations were the main subjects of the research hotspots, which show a low focus on the tools that can be utilized for generic populations [10]. PREM tools are based on patients' experiences while receiving care [11]. One cannot ignore the system improvements through a PREM tool as it is expected to ease out the process and make it mistake-proof [12]. In the tool, approximately 96.8% of patients reported feeling well-informed about their dressing procedures, which highlights that the communication is satisfactory and demonstrates the tool’s effectiveness in evaluating communication. This aligns with a recent study highlighting the role of clear communication in enhancing patient satisfaction and compliance [13].

In the survey, nearly all patients (98.6%) reported that the healthcare workers adhered to proper hand hygiene and infection control practices. This is an interesting finding, as the patients were also examining infection control practices, which have become more evident since the COVID-19 pandemic [13]. This finding is consistent with the emphasis on aseptic techniques in wound care. The tool emphasises the importance of strict adherence to infection prevention protocols, underscoring the significance of aseptic practices in wound care [14].

Patient education is the key to improvised health care delivery. The findings of only 57.7% patients receiving education on home care practices shows that, there is a notable need for improved education. Comprehensive education is vital for effective home wound care This finding is consistent with findings of Fereidouni Z et al. [15]. Different patients may have varying levels of understanding and different types of wounds. Hence, education at this level can address specific needs and concerns, making the information more relevant and actionable. While patients generally felt well-informed, education is needed, particularly regarding home care and infection indicators [16].

Morton et al. conducted a cross-sectional survey of Patient-Reported Outcome Measures (PROMs)/PREMs use among renal hospitals. They highlighted the challenge of insufficient staff resources in hospitals for not using patient engagement tools [17]. Standardized use of PROMs and PREMs in clinical practice enables quantitative measurement and continuous improvement of health outcomes in surgery while promoting patient-centered healthcare [18]. Future studies should focus on refining the PREM tool based on patient feedback and evolving clinical practices. Addressing current limitations and incorporating patient suggestions can enhance its effectiveness. Bull et al. conducted a systematic review and concluded that in most PREM tools, validity and reliability tests must be considered, supported with appropriate study designs [19,20]. As patient experiences become increasingly integral to measuring value in healthcare across services and systems internationally, it is critical that the experiential attributes of healthcare captured by PREMs are meaningful to patients. Thus, examining PREM content validation in the eyes of patients is critical [21]. Research should explore the tool’s applicability across different healthcare settings and wound types. Comparative studies across various countries and healthcare systems could validate its utility more broadly. Medication decisions were better by comparison because patients were more likely to report they were asked for input, and the cons of taking medication were discussed to a greater extent [22]. Combining PREMs with traditional quality indicators, such as wound healing rates and infection rates, could provide a more comprehensive assessment of wound care quality. This integration can support a more holistic approach to wound management.

Strengths and limitations

While PREMs help to address the challenge of incorporating patient-specific risk factors into wound management, they may not fully account for all variables affecting healing outcomes. The study’s male-dominated sample reflects the existing literature on wound prevalence but may introduce gender-based biases. Future research should aim for a more balanced demographic representation to enhance generalizability. Although validated in the Indian context, the PREM tool’s applicability to other cultural and clinical settings remains to be tested. Adapting the tool to diverse contexts could offer a broader understanding of patient experiences globally.

## Conclusions

The development and validation of the PREM instrument mark a significant advancement in incorporating patient perspectives into wound care. Its strong reliability and validity highlight its potential to enhance patient-centered care and improve clinical practices. Addressing existing challenges and expanding its application can further optimize wound therapy outcomes.

## Appendices

Patient-Reported Experience Measures (PREM) Questionnaire for Wound Management

Instructions:

Please answer the following questions based on your most recent experience with wound dressing. Your responses will help improve the quality of care provided in wound management.

Patient Information

Age (years): __________________

Gender: ☐ Male ☐ Female ☐ Other: _______________

Did you fill out this questionnaire? ☐ Yes (Patient) ☐ No (Bystander/Family Member)

Wound Dressing Procedure

Was your name and ID verified by the healthcare provider before the dressing procedure? ☐ Yes ☐ No

Was the procedure explained to you before it was started? ☐ Yes ☐ No

Were you informed about any discomfort or pain that could occur during the dressing procedure? ☐ Yes ☐ No

Did the healthcare provider ensure privacy during the dressing procedure? ☐ Yes ☐ No

Was personal jewelry removed as part of the procedure? ☐ Yes ☐ No ☐ Not Applicable

Did the healthcare provider follow hand hygiene procedures (e.g., handwashing or use of hand sanitizer)? ☐ Yes ☐ No

Infection Control

Did the healthcare team take infection control precautions (e.g., wearing gloves, using sterilized equipment)? ☐ Yes ☐ No

Patient Communication

Did you feel comfortable asking the healthcare provider questions about the procedure? ☐ Yes ☐ No

Was the wound management plan explained to you by the healthcare provider? ☐ Yes ☐ No

Patient Education

Were you instructed on how to care for your wound at home? ☐ Yes ☐ No

Were you able to demonstrate the wound dressing technique to the healthcare provider? ☐ Yes ☐ No

Were you advised to look for signs of infection (e.g., wetness, fever) in the dressing? ☐ Yes ☐ No

Satisfaction and Concerns

Were your concerns about the procedure addressed by the healthcare provider? ☐ Yes ☐ No

Were you informed about when to seek urgent care if necessary? ☐ Yes ☐ No

Do you know whom to contact for urgent wound-related issues? ☐ Yes ☐ No

Additional Comments :
